# Incidence of Traffic Accidents in Gimhae City, Republic of Korea 

**Published:** 2019-08

**Authors:** Jeongyee Bae, Hwan-sun Kim, JeongYeol Lee, Oe-gyeong Oh, Chang-Hyo Bae, Dong-su Han, Sung-Il Cho

**Affiliations:** ^*a*^Chair, Inje University Institute for International Safe Promotion and Professor, Inje University.; ^*b*^Public Official, Gimhae City, Republic of Korea.; ^*c*^Research Assistant, Inje University Institute for International Safe Promotion.

## Abstract

**Background:**

The number of deaths from traffic accidents in Korea has been steadily decreasing every year. ‎However, the death rate of traffic accidents in Korea is 8.1 per 100,000 people in 2017, which is ‎the highest among OECD member countries. In addition, the number of traffic accidents per ‎‎10,000 cars is 1.9, which is the highest among OECD member countries, and it is necessary to ‎have access to traffic safety. Gimhae city has three highway networks connecting to Gimhae ‎International Airport and it is a key role of transportation. Therefore, traffic volume and traffic ‎accident in Gimhae are relatively higher than other surrounding cities.Therefore, we need to ‎identify the incidence and causes of traffic accidents by age, place, time etc. Objective: The ‎purposes of this study are to identify the incidence of traffic accident in Gimge city and to ‎develop the evidence based traffic accident program for Gimhae city. ‎

**Methods:**

In order to understand the current state of traffic accidents for the citizens of Gimhae city, the ‎annual report on the causes of death statistics from the National Statistics Office in 2017 and the ‎annual report on traffic accidents from the National Police Agency in 2017 were analyzed. Base ‎on this data authors identified high risk factors related to traffic accidents such as traffic ‎accident occurrence rates, drinking and driving, seatbelts and other protective gears.‎

**Results:**

The death rate of traffic accidents in Gimhae city was 2.3 times higher in males than females. ‎And the rate of death has increased with aging, in the order of elderly (65 and over), middle-‎aged (45 to 64), adult (19 to 44), and youth (13 to 18). The rate of injuries caused by traffic ‎accidents was 3.4 times higher in males than females. Also, the rate of injuries increased with ‎aging, in the order of elderly (65 and over), middle-aged (45 to 64), adult (19 to 44), and youth ‎‎(13 to 18). There was the highest number of accidents in April especially on Thursdays. There ‎was the highest number of injury due to accidents during rush hour of 6PM to 10PM. As the ‎result of analyzing the incidence of drink-driving accidents according to the types of vehicles, ‎motorcycles, passenger cars, and car rentals were in the order. Seat belt attached rate were high ‎in the order of under 14 years old, 15-20 years and above 70 years. And helmet wearing rate ‎were high in the order of 15-20 years old, above 70 years and under 14 years old.

**Conclusions:**

Based on results of this study, it is desirable to develop customized traffic safety programs. In ‎the case of the elderly, there are a lot of pedestrian accidents, so we need program that can ‎improve the walking environment. For adult and middle-age, it will be effective to provide ‎drunk driving education and safety belt training programs. In terms of drink-driving preventive ‎education, we should be conducted for motorcycles and rental cars. For children and youth, it is ‎necessary to educate about wearing safety belts and helmets. Also, the traffic policies should be ‎strengthened for prevention of drunk driving. Therefore, the government and related ‎organizations should spread an effective evidence-based traffic accident program based on ‎result of this research.‎

**Keywords:**

Traffic accidents, Injury prevention, Safety programs

